# Prevalence of Potential Drug-Drug Interactions Among Hypertensive Pregnant Women Admitted to a Tertiary Care Hospital

**DOI:** 10.7759/cureus.36306

**Published:** 2023-03-17

**Authors:** Akshitha S Ragam, Sheela S R

**Affiliations:** 1 Obstetrics and Gynaecology, Sri Devaraj Urs Medical College, Kolar, IND

**Keywords:** rational drug use, clinically relevant, severity, hypertension in pregnancy, potential drug interactions

## Abstract

Aims and objectives: The aim is to determine the frequency of potential drug-drug interactions (pDDIs) and to analyze the clinically relevant drug interactions among hypertensive pregnant women.

Materials and methods: This was an observational, cross-sectional study conducted at a tertiary care hospital. The prescriptions of the hypertensive pregnant women admitted to the hospital from June 2021 to December 2021 were analyzed for potential drug-drug interactions using the database from Lexicomp ® Solutions android mobile application version 7.5.4 (Wolters Kluwer, The Netherlands).

Results: A total of 127 patients were evaluated during the study period of 6 months, of which 70 (55.12%) had pDDIs. The total number of pDDIs was 85, of which 70 (82.35 %) were clinically relevant interactions with the majority of them having moderate severity (81.17%) followed by minor severity (17.65%) and major severity (1.18%). The most frequently interacting pDDIs were between Labetalol and Lornoxicam (42.35%), followed by Labetalol and Diclofenac (22.35%).

Conclusion: This study highlights the high prevalence of potential drug interactions among hypertensive pregnant women and the need for rational drug use and strict vigilance in their monitoring.

## Introduction

A change in the therapeutic impact and safety of one or both pharmaceuticals as a result of the concurrent or subsequent administration of two different medications is known as a drug interaction. Changes in the medication's pharmacokinetics, which modify the drug's delivery to the site of action, or changes in the drug's pharmacodynamics, which vary the response of the drug targets, may lead to drug interactions [1]. Potential drug-drug interactions (pDDI) are caused by a variety of variables, including age, comorbid diseases, and the number of drugs administered [2]. According to several reports, the incidence of pDDIs in individuals using 3-10 medicines is between 3% and 5% [3]. According to Lima et al., patients taking 10-20 medications had a 10%-20% higher prevalence of pDDIs [4]. Drug interactions are the fourth most common cause of death, accounting for around 0.054% of visits to the emergency department, 0.57% of hospital admissions, 0.12% of readmissions, and 3%-4% of adverse drug reactions [5,6].

Prescription and administration of medications should be done with care due to the poorly known alterations in pharmacodynamics and pharmacokinetics caused by the physiological adaptations that happen during pregnancy [7]. Additionally, pregnant participants are often excluded from clinical trials due to ethical considerations, therefore knowledge of the safety of medication exposure during pregnancy is only available from post-marketing data generated from observational studies [8].

Parenteral magnesium sulphate, antihypertensives including calcium channel blockers, methyldopa, hydralazine, diuretics, and labetalol are all required as part of intense treatment for certain pregnancy issues, like hypertensive symptoms of pregnancy, such as pre-eclampsia and eclampsia [9-11].

Despite the rampant use of these medications, literature on the prevalence of drug interactions in such patients is lacking. Only clinical trials with carefully chosen patient groups or short case series have been noted in few published studies to investigate drug interactions related to magnesium sulphate [12-14].

As a result, this study was designed to determine the prevalence of pDDIs, to analyze clinically relevant pDDIs, record commonly used drug combinations, and classify them according to severity in order to increase clinicians' awareness and understanding in this field and reduce the negative effects caused by pDDIs.

## Materials and methods

The current study was an observational, cross-sectional investigation conducted at a tertiary care hospital. After receiving clearance from the Institutional Ethics Committee, one prescription from each hypertensive pregnant woman admitted to the Obstetrics and Gynaecology Department between June 2021 and December 2021 was gathered retrospectively and evaluated. A prescription within 48 hours after admission was chosen.

A proforma was filled out using the data where the demographic and patient identifying information, including the patient's age, diagnosis, parity, date of admission, and length of hospital stay, were included in Section 1. And the information on the recommended medications, including their name, dose, and frequency of administration, was included in Section 2. Using the database from the Lexicomp ® Solutions Android mobile application version 7.5.4 (Wolters Kluwer, The Netherlands) the following was done.

The drugs and pDDIs were classified and quantified according to the Anatomic Therapeutic Chemical (ATC) Classification, pDDIs were assessed for - severity: minor/moderate/major/contradicted, scientific evidence (documentation): fair/good/excellent, risk rating: A - No known interaction, B - No action needed, C - Monitor therapy, D - Therapy modification, X - Avoid combination and the frequently interacting drug combinations were analyzed.

The class of drugs that were included in the study were antibiotics, anti-hypertensives, analgesics, antipyretics, oral hypoglycemic agents, anti-thyroid drugs, synthetic hormones such as levothyroxine, steroids, proton pump inhibitors, anti-convulsant, antihistamines, anti-inflammatory drugs, diuretics, anti-coagulants, antiplatelet agents, anti-arrhythmic drugs, and inotropic agents. The study did not include substances like vitamins, hydro electrolytic components, or dietary supplements.

MS Excel and SPSS version 22 for Windows were used to conduct the descriptive statistical analysis. A “p” value of 0.05 or below was regarded as statistically significant. The Spearman correlation coefficient was used to assess the relationship between the frequency of pDDIs and age, hospital stay length, diagnoses made, and medications administered.

## Results

A total of 127 women were included during the six-month period; of these, 70 of them (55.12%) had pDDIs, with ages ranging from 18 to 40 years old and a mean of 25.16 ± 3.91 years (Table 1).

**Table 1:** Patient characteristics involved in drug interactions

Patient Characteristics	Mean 土 SD
Mean age	25.16 土 3.917
Mean number of days in hospital	10.08 土 3.273
Average number of diagnosis	2.19
Mean number of drugs prescribed per patient	4.93 土 0.782
Mean number of drug interactions per patient	1.27 土 0.426
Mean number of clinically relevant pDDIs	1.12 土 0.233

The total number of drugs administered was 627, with an average of 4.93 ± 0.78 medications per patient. As per the ATC Classification, the majority of the drugs prescribed belonged to the Cardiovascular system (26.72%), General anti-infectives (25.26%) and alimentary canal and metabolism (25.05%) (Table 2).

**Table 2:** Drug types according to ATC Classification and their prescription frequency ATC: Anatomic Therapeutic Chemical

ATC Classification	Drug Types (n)(%)	Prescription frequency (n)(%)
A- Alimentary tract & metabolism	1(3.22%)	120(25.05%)
B- Blood & blood forming organs	4(12.90%)	5(1.04%)
C- Cardiovascular system	8(25.80%)	128(26.72%)
G- Genitourinary System & sex hormones	1(3.22%)	1(0.20%)
H- Systemic hormonal prep, excluding sex hormones	4(12.90%)	18(3.75%)
J- General anti-infectives	5(16.12%)	121(25.26%)
M- Musculo-skeletal system	1 (3.22%)	4(0.83%)
N- Nervous System	4(12.90%)	74(15.44%)
R- Respiratory System	1(3.22%)	1(0.20%)
V- Various	2(6.45%)	7(1.46%)

There were 85 pDDIs in total, of which 70 (82.35%) were clinically significant interactions. 55.12% of the individuals had one or more pDDIs, with an average of one to four per subject while a majority of pDDIs were due to drugs associated with the cardiovascular system (88.23%) (Table 3).

**Table 3:** pDDIs according to ATC Classification and their frequency pDDIs: Potential Drug-Drug Interactions, ATC: Anatomic Therapeutic Chemical

ATC Classification	pDDI types (n(%))	pDDI frequency (n(%))
A- Alimentary tract & metabolism	-	-
B- Blood & blood forming organs	-	-
C- Cardiovascular system	7(53.84%)	75(88.23%)
D- Dermatologicals	-	-
G- Genitourinary system & sex hormones	1(7.69%)	1(1.17%)
H- Systemic hormonal prep, excluding sex hormones	3(23.07%)	6(7.05%)
J- Anti-infectives	-	-
L- Anti-neoplastic & immunomodulating agents	-	-
M- Musculo-skeletal system	-	-
N- Nervous system	1(7.69%)	2(2.35%)
R- Respiratory system	1(7.69%)	1(2.35%)
S- Sensory organs	-	-
V- Various	-	-

On the severity scale, most of the 85 pDDIs were of moderate severity with 81.17 %, while 17.65% were of minor severity and 1.18% were of major severity (Table 4). All the pDDIs with major and moderate severity were considered clinically relevant pDDIs which was 82.35%. Regarding the documentation of pDDIs, 72 (84.71%) of them had fair documentation in literature, followed by 11 pDDIs (12.94%) which had good documentation and two (2.35%) had excellent documentation. In risk rating, the majority of them, i.e., 67 (78.82%) pDDIs belonged to Category “C”and required monitoring of therapy. Fifteen (17.64%) belonged to Category “B” which required no further actions, two (2.35%) pDDIs had a risk rating of Category “D” which warranted therapy modification and one (1.17%) pDDI between Levosalbutamol and Labetalol fell into Category “X” whose combination was contraindicated as the β-blockers may antagonize the effects of β2 adrenergic bronchodilators precipitating acute, life-threatening bronchospasm.

**Table 4:** pDDIs according to their severity

		Frequency (n)	Percentage (%)
Potential Drug-Drug Interactions (pDDIs)	MILD	15	17.65
	MODERATE	69	81.17
	SEVERE	1	1.18

The most frequently interacting pDDIs were between Labetalol and Lornoxicam (42.35%), followed by Labetalol and Diclofenac (22.35%) in which the common probable mechanism was thought to be the diminishing effect of the anti-hypertensive effect of the β-blockers by the NSAIDs (Table 5).

**Table 5:** Important drug combinations leading to pDDIs pDDIs: Potential Drug-Drug Interactions, CCBs: Calcium Channel Blockers, NSAIDs: Non-Steroidal Anti-Inflammatory Drugs, MgSO_4_: Magnesium Sulphate

Sl no.	1st Drug	2nd Drug	Number (%)	Risk Rating	Severity	Documentation	Mechanism
1.	Labetalol	Lornoxicam	36 (42.35%)	C	Moderate	Fair	NSAIDs diminish the anti-hypertensive effect of β-blockers
2.	Labetalol	Diclofenac	19 (22.35%)	C	Moderate	Fair	NSAIDs diminish the anti-hypertensive effect of β-blockers
3.	Amlodipine	Diclofenac	5 (5.88%)	B	Minor	Excellent	NSAIDs diminish the anti-hypertensive effect of CCBs
4.	Amlodipine	MgSO4	4 (4.7%)	C	Moderate	Fair	MgSO4 may enhance the risk of toxic effect (hypotension/ muscle weakness) of CCBs
5.	Levosalbutamol	Labetalol	1 (1.17%)	X	Major	Fair	β-blockers may antagonize the effects of β2 adrenergic bronchodilators precipitating acute, life-threatening bronchospasm

**Figure 1:**
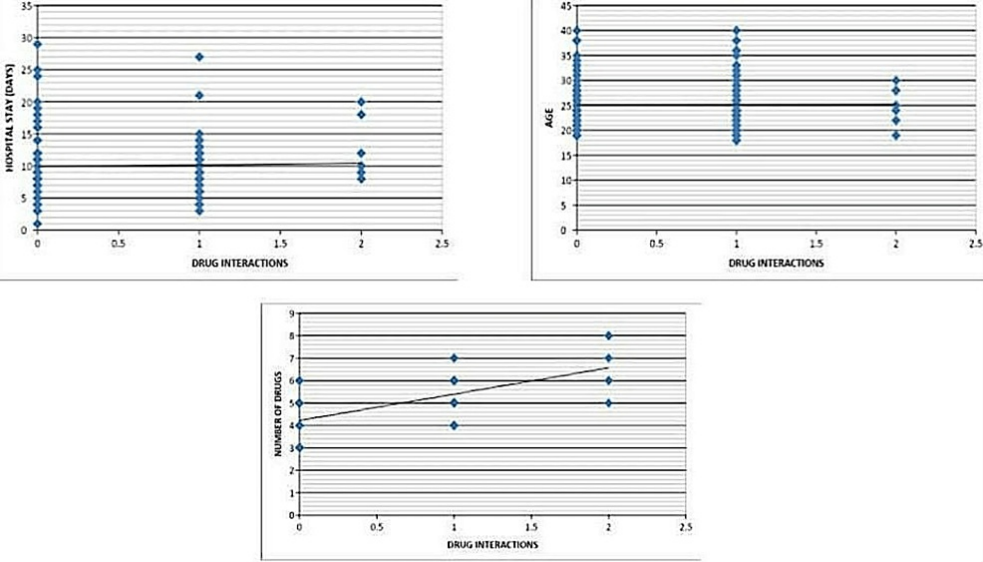
Correlation between age, number of drugs prescribed, number of days of hospitalization and pDDIs pDDIs: Potential Drug-Drug Interactions

Age, the number of medicines used, and the number of days spent in the hospital all had positive correlations with pDDIs of +0.006 (p=0.946), +0.637 (p=0.0001), and +0.034 (p=0.701), respectively. However, only the association between pDDIs and the number of drugs was statistically significant (Table 6, Figure 1).

**Table 6:** Correlation between age, number of drugs prescribed, number of days of hospitalization and pDDIs pDDIs: Potential Drug-Drug Interactions

		Hospital Stay	Age	No. of Drugs prescribed
Potential Drug-Drug Interactions	Pearson Correlation	0.034	0.006	0.637
	P-value	0.701	0.946	< 0.001

## Discussion

It has been documented that there is a correlation between the number of prescribed medications and the number of pDDIs. This association highlights the inherent danger of prescribing a variety of medications [15,16]. Although several studies have supported the medical treatment of pregnant women with severe chronic hypertension, it is unclear whether pregnant women with mild to moderate chronic hypertension should also get medical care^ ^[17]. Our study shows that there was a positive correlation between drug interaction with a hospital stay, age, and number of drugs with a statistically significant correlation only between the number of drugs and drug interactions (P<0.05).

Similar to the outcomes of other studies, a positive association between the number of prescription medications and diagnoses and the number of pDDI was also seen in our study [18,19]. A rise in diagnoses leads to an increase in medications given, which raises the likelihood of drug interactions and changes in therapeutic response.

About 85.4% of the interactions were significant interactions, as opposed to 73.37% in the study conducted by Patel et al. and 67.3% in the study by Chelkeba et al. The percentage of minor interactions in the current research was only 14.6%, whereas it was reported to be 22.9% by Patel et al. and 3.1% by Chelkeba et al. [20,21].

In this study, the severity of pDDIs was mild in 15 (17.6%), moderate in 69 (81.1%), and severe in one (1.3%), which was consistent with earlier studies [18]. According to a study by Pessoa et al. published in 2019, nearly one-third of pregnant women experienced at least one severe pDDI and were subjected to more than two-thirds of the hospital stay. A significant number of pharmaceuticals provided during the hospitalization period and past medication usage before admission were shown to be risk factors for severe pDDIs, which were present in around 33.9% of patients [22].

The method of interaction includes a decrease in flow in the calcium channels in myocardial cells as well as inhibiting norepinephrine production from the adrenal glands, thus counteracting the vasopressor effects [23]. For these patients to get the best treatment, the medical provider must be able to differentiate between drug interactions and the onset of signs when medical intervention is necessary to address the consequences. A strict level of vigilance is necessary while taking medications causing this type of interaction, where effects could lead to death, hospitalization, therapeutic failure, or permanent injury.

The true impact of these pDDIs should also be assessed on an individual basis, which necessitates the careful analysis of the risk-benefit relationship between stopping the medication and continuing it with ongoing monitoring. Most management best practices adopt this strategy and always conduct a risk-benefit analysis [24].

## Conclusions

The current study highlights the high prevalence of potential drug interactions among hypertensive pregnant women which were mostly moderate in severity, with comorbidities and polypharmacy being important determinants. Due to the increasing number of diagnoses and concurrent diseases, polypharmacy is a prevalent issue in pregnant women, necessitating extra safety precautions to monitor and avoid pDDIs. The occurrence of drug interactions may be decreased with the use of suitable software or with the help of a clinical pharmacist in charge of keeping an eye on drug interactions and alerting a physician about possible issues. Additional clinical correlation studies are required, and an education program for medical professionals is required to increase awareness and improve therapeutic regimens to provide better healthcare quality.
